# Microbial Sensing and Removal of Heavy Metals: Bioelectrochemical Detection and Removal of Chromium(VI) and Cadmium(II)

**DOI:** 10.3390/molecules26092549

**Published:** 2021-04-27

**Authors:** Reham A. Alfadaly, Ashraf Elsayed, Rabeay Y. A. Hassan, Ahmed Noureldeen, Hadeer Darwish, Ahmed S. Gebreil

**Affiliations:** 1 Botany Department, Faculty of Science, Mansoura University, Elgomhouria St., Mansoura City 35516, Egypt; rehamalfadaly123@yahoo.com (R.A.A.); bio_botany@yahoo.com (A.S.G.); 2 Nanoscience Program, University of Science and Technology (UST), Zewail City of Science and Technology, 6th October City, Giza 12578, Egypt; ryounes@zewailcity.edu.eg; 3 Applied Organic Chemistry Department, National Research Centre (NRC), Dokki, Giza 12622, Egypt; 4 Department of Biology, College of Sciences, Taif University, P.O. Box 11099, Taif 21944, Saudi Arabia; a.noureldeen@tu.edu.sa; 5 Department of Biotechnology, College of Sciences, Taif University, P.O. Box 11099, Taif 21944, Saudi Arabia; hadeer@tu.edu.sa

**Keywords:** biosensing of heavy metal contaminants, microbial electrochemistry, biofilm formation, hexavalent chromium, Cr(VI)

## Abstract

The presence of inorganic pollutants such as Cadmium(II) and Chromium(VI) could destroy our environment and ecosystem. To overcome this problem, much attention was directed to microbial technology, whereas some microorganisms could resist the toxic effects and decrease pollutants concentration while the microbial viability is sustained. Therefore, we built up a complementary strategy to study the biofilm formation of isolated strains under the stress of heavy metals. As target resistive organisms, *Rhizobium*-MAP7 and *Rhodotorula* ALT72 were identified. However, *Pontoea agglumerans* strains were exploited as the susceptible organism to the heavy metal exposure. Among the methods of sensing and analysis, bioelectrochemical measurements showed the most effective tools to study the susceptibility and resistivity to the heavy metals. The tested *Rhizobium* strain showed higher ability of removal of heavy metals and more resistive to metals ions since its cell viability was not strongly inhibited by the toxic metal ions over various concentrations. On the other hand, electrochemically active biofilm exhibited higher bioelectrochemical signals in presence of heavy metals ions. So by using the two strains, especially *Rhizobium*-MAP7, the detection and removal of heavy metals Cr(VI) and Cd(II) is highly supported and recommended.

## 1. Introduction

There are a number of pollutants and toxic agents, such as fertilizers, pesticides, and heavy metals, which extremely disturb the living and non-living systems. Heavy metals, such as Hg, Pb, Cr and Cd, are causing specific toxicity symptoms even in low concentrations of about 1.0–10 mg/L because they are being accumulated in the soft tissues [1,2,3,4]. The toxicity of heavy metal may result from alterations of numerous physiological processes caused at cellular/molecular level by deactivating enzymes functions, blocking active sites or functional groups of metabolically significant molecules, displacing or substituting for essential elements and disrupting membrane integrity [5,6,7]. Cr(VI) is carcinogenic and toxic even in small amounts which diffuses through the epidermis and reduces to Cr (III) that interacts with nuclear enzymes, proteins nucleotides and DNA [8]. Ingestion of Cr ions in large amounts can cause stomach upsets and stomach ulcers, convulsion, kidney and liver damage and even death [9]. On the other hand, cadmium is extremely toxic to humans by bioaccumulation in the kidney and the liver through the food chain [10,11]. The level of cadmium and chromium should not be beyond the permissible limit, ≤0.003 mg/L, and ≤0.05 mg/L, respectively [12].

For detecting acute toxicity caused by heavy metals, pesticides and organic solvents, several biochemical assays were applied based on the function of microorganisms [13], bacteria [14], antibodies [15] and enzymes [16]. Indicator microbes provide a simpler method [17] because microorganisms have biosorption capabilities as well as they are easy to culture in a short generation time so their response to toxic substances is quite rapid [18]. Soil bacteria *Rhizobium* sp. is one of the major elements for the maintenance of soil fertility where it has the ability to fix nitrogen in leguminous plants [19,20,21]. Symbiotic nitrogen fixation is sensitive to heavy metals in soil [22]. *Rhizobium* can be used as an indicator organism to several toxic chemicals, including heavy metals [23] and for effective, economical and eco-friendly metal bioremediation technologies [24] whereas *Sinorhizobium meliloti* has high tolerance ability for various heavy metals [25]. Cell wall components of microorganisms and pigments could have active metal sorption sites that are able to accumulate cadmium and lead simultaneously [26,27,28,29]. Amongst biosensors used techniques for online evaluation of microbial activity and intra/extracellular functions, microbial electrochemical systems (MESs) were developed and applied in many applications [30,31,32,33,34,35].

The microbial electrochemical-based biosensor can lead to a cost-effective, simple and repeatable measurement, which can provide rapid screening of heavy metals and their toxic effects in water-based environments. Thus, this work aims to develop microbial electrochemical systems that support the rapid detection for the microbial removal of Cr(VI) and Cd(II) using a selection of viable microbial cells.

## 2. Results and Discussion

### 2.1. Effect of the Metal Ions on the Growth and Cell Viability of the Selected Microbes

**Figure 1:**
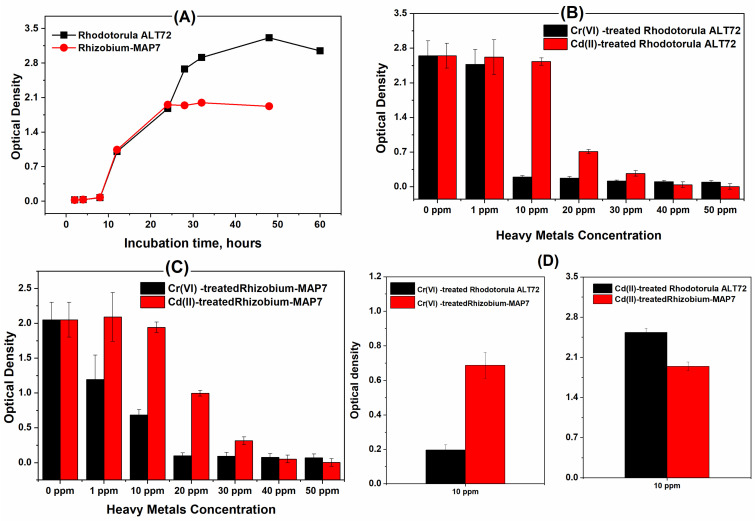
(**A**) Typical growth curves of untreated cells of *Rhodotorula* ALT72 and *Rhizobium*-MAP7, (**B**) effect of metal ions concentrations on the growth inhibition of *Rhodotorula* ALT72, (**C**) sensitivity of *Rhizobium*-MAP7 to Cr(VI) and Cd(II) different concentrations and (**D**) responses of *Rhodotorula* ALT72 and *Rhizobium*-MAP7 to a single concentration of Cr(VI) and Cd(II). The results recorded after 48 h for the *Rhizobium*-MAP7 and 60 h for the *Rhodotorula* ALT72.

Classical monitoring of the untreated cells of the *Rhodotorula* ALT72 and *Rhizobium*-MAP7 showed that the *Rhodotorula* ALT72 has faster growth rate with higher biomass production, whereas the stationary state was observed after 30 h (Figure 1A). On the other hand, resistivity or sensitivity of the selected microbes towards the toxic effects of chromium and cadmium ions was studied among several concentrations of the metal ions (0, 1, 10, 20, 30, 40 and 50 mg/L). Normal growth was observed at the low concentration, while the growth inhibition was obtained when the concentration was exceeded 10 mg/L for chromium, and 20 mg/L for cadmium (As can be depicted from Figure 1B,C). Taking into consideration, chromium and cadmium have different toxic effects on the selected two strains. In that sense, *Rhizobium* was less sensitive to the toxic effects of chromium(VI), as can be seen from Figure 1D.

As is shown in (Table 1), both of the *Rhodotorula*-ALT72 and *Rhizobium*-MAP7 were exposed to several concentrations of both metal ions, while the uptake rate of heavy metal was analyzed by considering the remaining concentration in the supernatants of the microbial culture. At the highest dose, i.e., the lethal concentration which is 50 mg/L, *Rhodotorula*-ALT72 still survived and showed an uptake of Cd ions with 1.274%. However, the *Rhizobium*-MAP7 did not show any capacitance of removal at this concentration. Regardless the lethal concentration, *Rhizobium*-MAP7- has higher removal efficiency than the *Rhodotorula*-ALT72, whereas the highest removal efficiency was reached 75% by the *Rhizobium*-MAP7 compare to 63.5% by the *Rhodotorula*-ALT72 at the concentration of 0.1 mg/L. In the essence of chromium(VI) removal, the result shown in Table 1 exhibited the higher efficiency of the Cr(VI) by the *Rhizobium*-MAP7 whereas the maximum capacity of removal was about 45% at the concentration of 0.1 mg/L. Nevertheless, both strains could not survive at the lethal dose; therefore, zero-% of removal was attained.

### 2.2. Testing the Cell Responses to the Metal Ions

**Figure 2:**
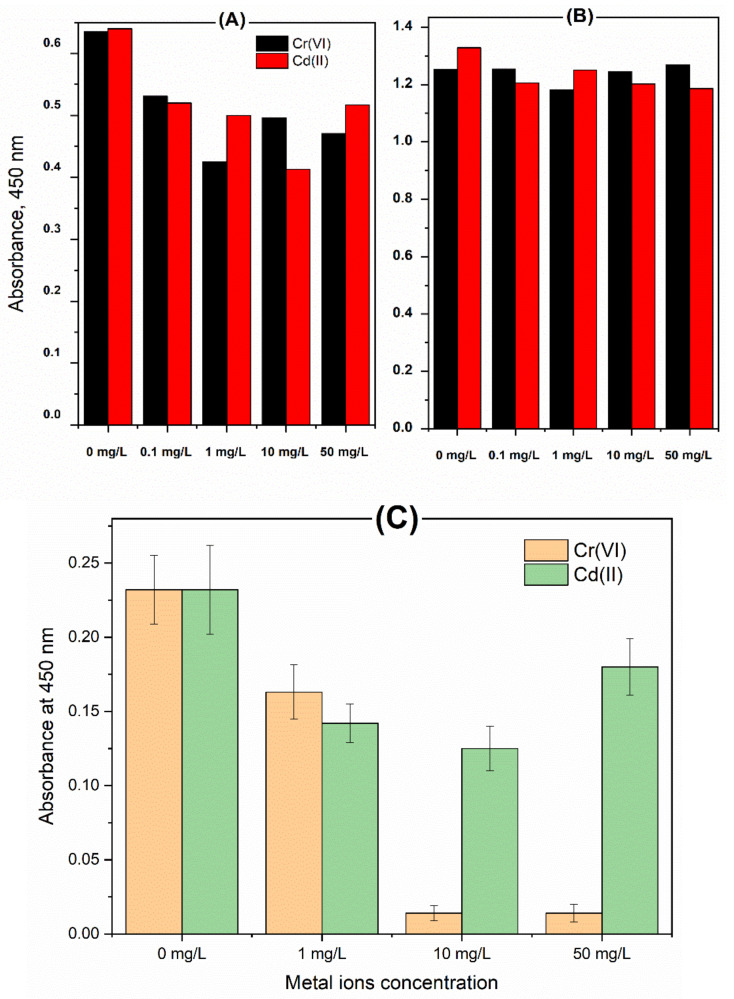
Testing the cell viability of treated *Rhodotorula* ALT72 (**A**) and *Rhizobium*-MAP7 (**B**) using WST-test and (**C**): testing the cell viability of *Pantoea agglomerans* using WST-test.

Usually, toxic effects of metal ions on the microbial activity are detected by monitoring the inhibition in the growth rate. Nevertheless, following up the changes in cell populations is a tedious and time-consuming process. Hence rapid screening of toxic effects on the cell viability is highly desirable. Therefore, a quick cell viability assay was here implanted using the WST which measures the metabolic activity along with the electron transport chain efficiency, [36]. Accordingly, *Rhodotorula* ALT72 and *Rhizobium*-MAP7 were treated with different concentrations of Cr(VI) or Cd(II) (0.1, 1, 10 and 50 mg/L). As depicted in Figure 2A, *Rhodotorula* ALT72 was more sensitive to the heavy metals than the *Rhizobium*-MAP7, since its cell viability was strongly inhibited using several concentrations. Nevertheless, the resistivity of the *Rhizobium*-MAP7 to the heavy metals was obvious as the cell viability was not affected after the heavy metal treatments, as can be shown in Figure 2B. Surprisingly, chromium ions were lethal even at the moderate concentration (1 mg/L) when a sensitivity bacterial strain was treated. This result was demonstrated in Figure 2C. Thus, the use of the *Rhizobium*-MAP7 as a target strain for the high-affinity detection and capture of heavy metal contaminants is achieved to the current extent.

### 2.3. Biosensing the Microbial Response to Heavy Metals Exposure

**Figure 3:**
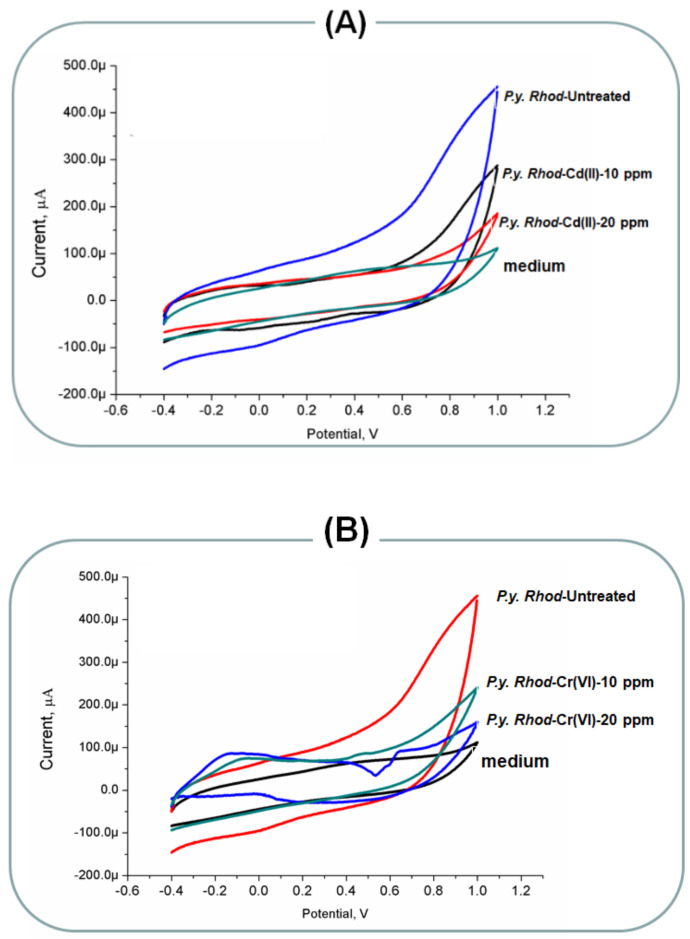
(**A**) Bio-electrochemical responses of biofilm formed *Rhodotorula* ALT72 treated with different concentrations of Cd(II). The untreated cells were considered as the positive control and (**B**) bio-electrochemical responses of biofilm formed *Rhodotorula* treated with different concentrations of Cr(VI). The untreated cells were considered as the positive control.

Microbial electron transport chain (METC) represents the most important compartment in the living systems, since the oxidation of degradable organic substrates is the main energy source of live microbial cells [37]. Therefore, measuring the efficiency of microbial respiration and the activity of the electron transport chain are considered main indicators of cellular activity, as they are essential for the replication and proliferation of aerobic organisms [38]. Consequently, the electron transfer process from living-microorganisms towards electrodes in microbial electrochemical systems (MESs) is exploited in microbial fuel cells or diagnostic tools for rapid assessment of microbial activity [39,40,41]. In these regards, many MES approaches were designed and tested for biological purposes [42,43,44,45]. The electrical current value generated by the MESs is directly proportional to the number of viable microbial cells that are incorporated in MESs. On the other hand, non-viable or non-cultivable living cells do not have electrochemical contribution, and thus, they do not generate electrochemical signals. Thus, the resulting bioelectrochemical responses reflect the extent of anodic respiration, intracellular redox reactions (e.g., intracellular enzyme activities) and/or other biological interactions [46,47]. Since the bioelectrochemical responses can be linked to microbial processes, the design of high-performance MESs has gained increasing attention due to their many promising applications in the environment, energy and biomedical fields [48]. Formation of biofilms at the sensor’s surface has been used for determining the microbial responses to the toxic effects of the utilized heavy metals. The main idea behind the bioassay is the receiving of electrons directly from the colonized microorganisms on the conductive sensors surfaces. The obtained curves are known as voltammograms, whereas the generated electric current is expressing the number of viable cells participating in the bioelectrochemical reactions. In this regard, any decrease in the generated current is referring to the encountered toxic effects. To that end, the selected strained were treated with the metal ions and were incubated with the sensors for two weeks in order to allow the formation of biofilms at the sensor’s surface. Accordingly, bioelectrochemical performances were analyzed. The results revealed in Figure 3 demonstrated the decrease in the activity of the biofilm supported by the electrode surfaces due to the existence of metal ions in the microbial culture. As displayed in Figure 3A, the interactions or the communication between the targeted bacterial and the sensor’s surface have been measured in presence of two different concentrations (10 and 20 mg/L) of the Cr(VI) and Cd(II). To that end, lower electrochemical signals were acquired from the cell of *Rhodotorula*-ALT72 treated with cadmium concentration. Nevertheless, the untreated cells of *Rhodotorula*-ALT72 produced higher electrochemical signals, i.e., about two-fold increase in the electrochemical readouts. On the other hand, Figure 3B demonstrated the voltammetric responses of chromium-treated vs the untreated cells of *Rhodotorula*-ALT72 ions. Pronounced increase in the voltammetric signals were obtained from the untreated cells, and the inhibition of the electrochemical signals were dependent on the heavy metal concentrations.

### 2.4. Rhizobium Bioelectrochemical Performance

**Figure 4:**
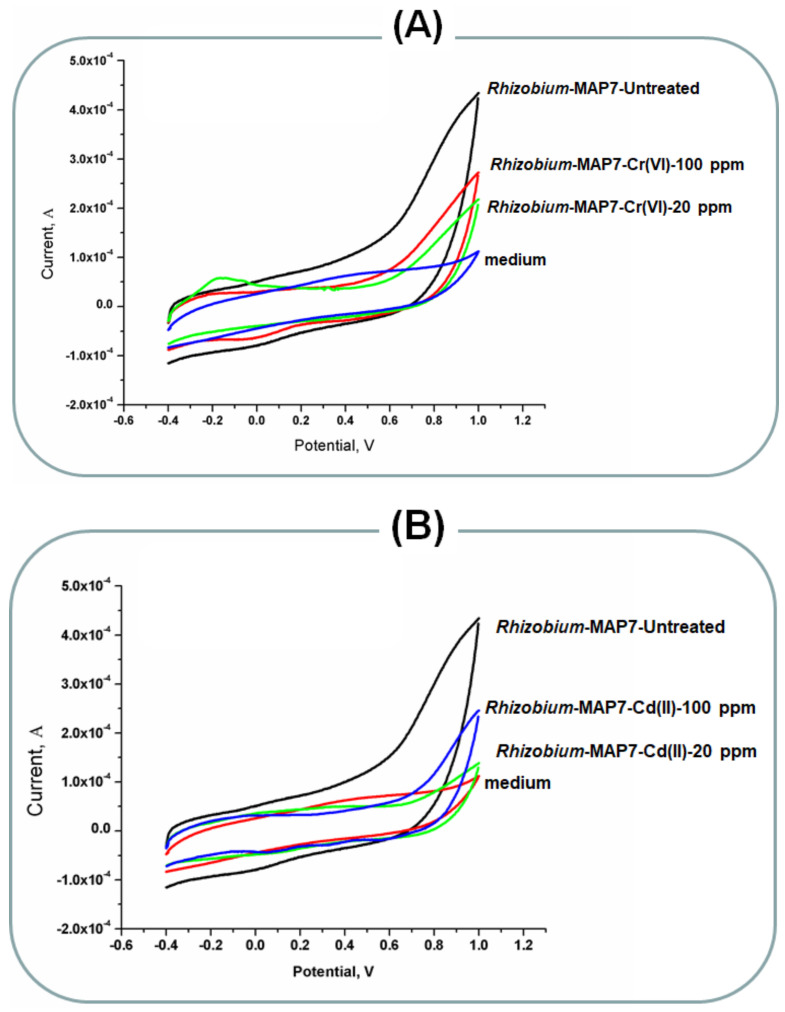
(**A**) Bio-electrochemical responses of biofilm formed *Rhizobium*-MAP7 treated with different concentrations of Cr(VI). The untreated cells were considered as the positive control and (**B**) bio-electrochemical responses of biofilm formed *Rhizobium*-MAP7 treated with different concentrations of Cd(II). The untreated cells were considered as the positive control.

Following the same manner, the interaction of *Rhizobium*-MAP7 with the MnO_2_ nano-rods were measured in presence of two different concentrations (10 and 20 mg/L) of the Cr(VI) and Cd(II). To that effect, the voltammetric signals of the treated cells were much lower than obtained by the untreated cells, as can be figured out from Figure 4. However, the toxic effects were more strongly sounding here if we compare these results with those obtained from the *Rhodotorula*. These findings revealed the stronger sensitivity of *Rhodotorula* to the toxic effects of the tested heavy metals.

**Figure 5:**
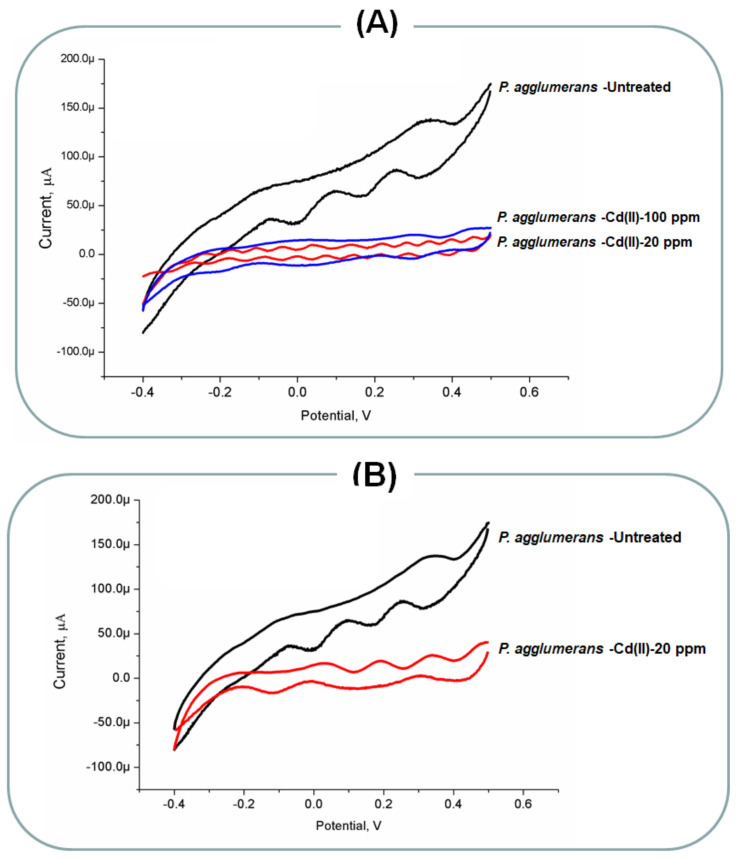
(**A**) Bio-electrochemical responses of biofilm formed *P. agglumerans* treated with different concentrations of Cd(II). The untreated cells were considered as the positive control and (**B**) bio-electrochemical responses of biofilm formed *P. agglumerans* treated with Cr(VI). The untreated cells were considered as the positive control.

*P. agglumerans* biofilm formation and bioelectrochemical responses to Cr(VI) and Cd(II) ions. To show the big difference between the sensitive and resistive of microbial strains towards the toxic effects of heavy metals, *P. agglumerans* was treated with cadmium, and chromium ions and their bioelectrochemical performances were put in a comparison with the above discussed two microbial strains (the *Rhizobium* and the *Rhodotorula*). The obtained results were amazing (As shown in Figure 5), since the treated cells with the moderate concentrations of heavy metals did not respond electrochemically, and their electrode interaction was not detected. Worthwhile, the untreated culture of *P. agglumerans* was responding efficiently.

Previously, *Rhizobium* has been used as an indicator organism to several toxic chemicals including metallothioneins that can bind to metals ions through the thiol group of its cysteine residues [49,50,51]. On the other side, *Rhodotorula* showed the potential remove ions of heavy metals [52]. To that event, heavy metals (Hg (II), Cu(II), and Pb(II) on the *Rhodotorula mucilaginosa* biofilm and planktonic cells was reported. In that study, minimum inhibitory concentration, minimum lethal concentration, as well as the minimum biofilm eradication concentration of the *R. mucilaginosa* were determined. The efficiency of heavy metal removal by planktonic cells from *R. mucilaginosa* was compared to the metal tolerance and removal efficiency by the biofilm. As they found in their study, biofilm tolerance was higher than the planktonic cells [52]. Thus, the use of biofilm formation for heavy metal removal and tracking the changes of metal ions concentration on-site was the main focus of our report with including two other heavy metal ions (i.e., the chromium and cadmium).

Biosensors and microbial electrochemical systems are currently exploited for heavy metal determination such as mercury, silver, copper, cadmium, lead, chromium and nickel [48,53]. From the microbiologically point of view, the existence of heavy metal pollutants in the medium can affect the electrochemically active microbes’ metabolic activity, leading to decreased transfer of electrons and weak present manufacturing. Therefore, microbial electrochemical systems were applied for online monitoring the toxic effects on microbes [54].

## 3. Materials and Methods

### 3.1. Microorganisms and Growth Conditions

Gram-negative, nitrogen fixer soil bacterium *Rhizobium*-MAP7 was obtained from the Physiology Laboratory at the Faculty of Science, Mansoura University, Mansoura, Egypt. Unicellular pigmented yeasts *Rhodotorula* ALT72, was obtained from the Cell and Genetics Laboratory, Faculty of Science, Mansoura University. Both strains were grown in L.B medium at 28 ± 1 °C with shaking at 150 rpm, while the *P. agglumerans* bacteria were grown in the L.B medium at 37 °C, obtained from Bacteria Laboratory, Faculty of Science, Mansoura University, Mansoura, Egypt, were used as controls. The growth rate estimated by measuring the optical density at wavelength 600 nm after 48 h of incubation for *Rhizobium*-MAP7, after 60–72 h for *Rhodotorula* ALT72 and after 24 h for *P. agglumerans*.

### 3.2. Determination of Toxic Effects and the Minimum Inhibitory Concentration

Sterilized LB media amended with different concentrations (0, 1, 10, 20, 30, 40 and 50 mg/L) of Cr(VI) or Cd(II) were inoculated with 100 µL of the overnight cultures of *Rhizobium*-MAP7 or *Rhodotorula* ALT72 (individual treated flasks for each strain) at 28 ± 1 °C, 150 rpm. To monitor the growth inhibition, optical density of each flask was determined photometrically at 600 nm after 48 h for the *Rhizobium*-MAP7 and 60 h for the *Rhodotorula* ALT72.

### 3.3. Heavy Metals Analysis

One hundred ppm of both chromium(VI) and cadmium(II) standard solutions were prepared from their potassium dichromate (K_2_Cr_2_O_7_), and cadmium nitrate salt, respectively. Concentrations of Cr(VI) and Cd(II) were determined in bacterial culture supernatants by atomic absorption spectroscopy. Supernatants of the treated cells with various concentrations (0.01, 0.1, 1, 10 and 50 mg/L) of the metal ions were collected by centrifugation after different incubation times. The remaining concentrations of heavy metals in supernatants were compared with the basic concentrations of heavy metals before inoculation. The results were expressed as percentage of metal removal by using the following equation:

(1) $$\mathrm{Metal}\;\mathrm{ions}\;\mathrm{removal}\;\left(\%\right)=\frac{\mathrm{Ci}-\mathrm{Cf}}{\mathrm{Ci}}\times 100$$

where, Ci and Cf stand for the initial and final heavy metal concentrations respectively.

### 3.4. Testing the Cell Proliferation Using WST-Test

Proliferation along with the response of bacterial and fungal cell suspension towards the toxic effects has been determined colorimetrically using the chemical reagent of water-soluble-tetrazolium salt (WST). In test tubes, three ml of sterilized LB media has been amended with different concentrations (0.1, 1, 10 and 50 mg/L) of both Cr(VI) and Cd(II) metal ions. Then, one mL inoculum culture of the *Rhizobium*-MAP7, *Rhodotorula* ALT72 and *P. agglumerans* were incubated under shaking for 30 min at 28 ± 1 °C for (*Rhodotorula* ALT72 and *Rhizobium*-MAP7) and 37 °C for *P. agglumerans*. Accordingly, a solution of the water-soluble-tetrazolium salt (WST) reagent has been added to each test tube (i.e., 20 μL from a stock solution of 10 mM). Afterwards, the absorbance at 450 nm has been measured and reflected as a zero time. Lastly, the stained microbial cultures have been incubated for further 30 min prior detecting the yellow color intensity that represents the cell viability and metabolic activity [39]. The inhibition in cell viability has been calculated according to the following formula:

(2) $$\mathrm{Percent}\;\mathrm{of}\;\mathrm{inhibition}=\frac{\mathrm{color}\;\mathrm{response}\;\mathrm{of}\;\mathrm{treated}\;\mathrm{cells}}{\mathrm{color}\;\mathrm{response}\;\mathrm{of}\;\mathrm{untreated}\;\mathrm{cells}}\times 100$$

### 3.5. Susceptibility of Microorganism to Heavy Metals

Cultures of *P. agglomerans* were treated with different concentrations of Cr(VI) and Cd(II), and incubated for 24 h at 37 °C, with shaking at 150 rpm. The optical density of each flask was determined at 600 nm.

### 3.6. Biofilm Formation and Bioelectrochemical Measurements

All the microbial electrochemical investigations have been conducted with a voltammetric setup using a platinum wire as the counter electrode, a Ag/AgCl/3M KCl as the reference electrode, and a MnO_2_-modified carbon electrode as the working electrode. The Gamry Potentiostat/Galvanostat/ZRA G750 system has been used for recording and analysis of the electrochemical assays. To investigate the response of microbial cells to different heavy metals, a suspension of *Rhodotorula* ALT72, *Rhizobium*-MAP7 and *P. agglumerans* as control were incubated with different concentrations of Cr(VI) and Cd(II) for two weeks to form mature biofilms at the working electrode surfaces. Consequently, at different time intervals, the electrochemical responses of biofilms to the heavy metals was recorded continuously to monitor the online progression of biofilms at the surface of the modified electrodes [55,56].

## 4. Conclusions

According to the data obtained in this study, *Rhodotorula*-ALT72 and *Rhizobium*-MAP7 can survive under stress of heavy metals ions Cr(VI) and Cd(II), while the chromium ions are more toxic than cadmium ions to both strains and also could be removed with higher percentage under concentrations 0.1 and 1 mg/L. Bioelectrochemical systems were used effectively to study the dynamic changes and cellular responses to the toxic metal ions. *Rhizobium*-MAP7 provides higher ability of removal of heavy metals and more resistive to metals ions since cell viability was not inhibited by either the toxic metal ions over the various concentrations than *Rhodotorula*-ALT72. Moreover, the faradic currents which result from electrochemically active biofilm in presence of heavy metals ions is higher in *Rhodotorula*-ALT72 than *Rhizobium*-MAP7 while the untreated cells were still producing higher electrochemical signals. So using the two strains especially *Rhizobium*-MAP7 support the detection and removal of heavy metals Cr(VI) and Cd(II).

## Figures and Tables

**Table 1:** Measuring the remaining concentrations of metal ions in the microbial supernatants after culturing the *Rhodotorula* ALT72 and *Rhizobium*-MAP7 in synthetic contaminated liquid media. Metal ions concentrations were detected by Atomic Absorption Spectroscopy.

Heavy Metals Conc.		*Rhodotorula* sp.		*Rhizobium* sp.	
Type	Initial Concentration mg/L	Remaining Concentration mg/L	Removal %	Remaining Concentration mg/L	Removal %
Cr(VI)	0.01	0.0064	36.00%	0.0056	44.00%
	0.10	0.085	14.70%	0.055	44.90%
	1.00	0.95	5.20%	0.85	15.03%
	10.00	9.5	5.07%	9.73	2.70%
	50.00	50.00	0.00%	50.00	0.00%
Cd(II)	0.01	0.0084	63.00%	0.0048	52.00%
	0.10	0.0365	37.50%	0.025	74.70%
	1.00	0.626	16.39%	0.51	48.93%
	10.00	9.85	1.45%	9.08	9.21%
	50.00	49.87	1.27%	50.00	0.00%

## Data Availability

The data presented in this study are available in within the article.

## Sample Availability

Sample of the compounds are not available from the authors.
